# Correction to: Debottlenecking the L-DOPA 4,5-dioxygenase step with enhanced tyrosine supply boosts betalain production in Nicotiana benthamiana

**DOI:** 10.1093/plphys/kiae361

**Published:** 2024-08-02

This is a correction to: Soyoung Jung, Hiroshi A Maeda, Debottlenecking the L-DOPA 4,5-dioxygenase step with enhanced tyrosine supply boosts betalain production in *Nicotiana benthamiana*, *Plant Physiology*, 2024; https://doi.org/10.1093/plphys/kiae166

In the originally published version of the manuscript section C of Figure 5 was omitted in error.

The article has now been emended to include this section.

